# Real-time current of injury monitoring for safe Bachmann bundle lead fixation

**DOI:** 10.1093/ehjcr/ytag500

**Published:** 2026-07-17

**Authors:** Takashi Ikee, Kohei Ishibashi, Nobuhiko Ueda, Kengo Kusano

**Affiliations:** Department of Cardiovascular Medicine, National Cerebral and Cardiovascular Center, 6-1 Kishibe-Shimmachi, Suita, Osaka 564-8565, Japan; Department of Cardiovascular Medicine, National Cerebral and Cardiovascular Center, 6-1 Kishibe-Shimmachi, Suita, Osaka 564-8565, Japan; Department of Cardiovascular Medicine, National Cerebral and Cardiovascular Center, 6-1 Kishibe-Shimmachi, Suita, Osaka 564-8565, Japan; Department of Cardiovascular Medicine, National Cerebral and Cardiovascular Center, 6-1 Kishibe-Shimmachi, Suita, Osaka 564-8565, Japan

## Case description

A 78-year-old man underwent balloon-expandable transcatheter aortic valve implantation for severe aortic stenosis. Postoperatively, he developed symptomatic paroxysmal complete atrioventricular block requiring permanent pacemaker implantation. Given the patient’s history of paroxysmal atrial fibrillation, Bachmann bundle pacing was selected to maintain atrial synchrony and improve interatrial conduction.

A SelectSecure 3830 pacing lead (4.1 Fr, 69 cm; Medtronic, MN, USA) was delivered using a fixed-curve C315-S502 sheath (Medtronic) and positioned at the superior interatrial septum. During screw advancement, the COI was continuously monitored in real time (*Figure A*) using the CareLink SmartSync™ device manager (Medtronic). Current of injury was observed by unipolar sensing before screw advancement and increased gradually during the first three rotations. At five rotations, the COI jumped up abruptly, with a waveform markedly different from baseline (*Figure B*), suggesting stable myocardial engagement and fixation. Subsequent bipolar pacing demonstrated a paced P wave shortened by more than 10 ms compared with the intrinsic P wave, with increased amplitude and sharper morphology (*Figure C*), consistent with electrophysiological criteria for Bachmann bundle capture. At implantation, the atrial sensing amplitude was 1.6 mV, the pacing threshold was 0.5 V at 0.4 ms, and the lead impedance was 665 Ω. The procedure was completed without complications, and lead parameters remained stable at 4-month follow-up (*Figure D*). This case highlights the utility of COI-guided fixation in high-risk atrial pacing sites.

**Figure:**
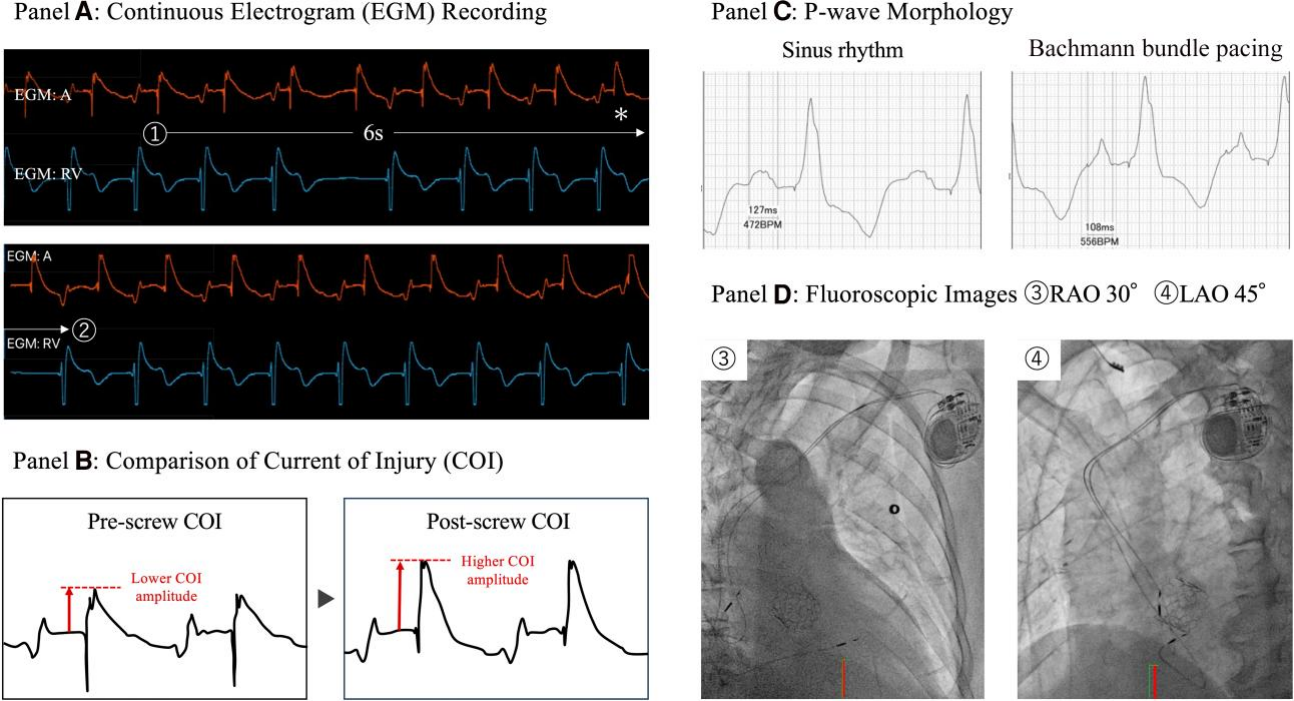
Real-time COI monitoring during Bachmann bundle lead fixation. (*A*) Continuous EGM recording obtained during active fixation of the atrial lead in the Bachmann bundle region. ① indicates three lead rotations and ② indicates five lead rotations. During the fifth lead rotation (*), an abrupt change in COI morphology was observed, followed by an increase in COI amplitude. (*B*) Comparison of COI recordings before and after lead fixation. The post-screw recording demonstrated a higher COI amplitude than the pre-screw recording. (*C*) Comparison of P-wave morphology during sinus rhythm and Bachmann bundle pacing. Bachmann bundle pacing resulted in a higher-amplitude P wave and a P-wave duration shortened by more than 10 ms. (*D*) Fluoroscopic images in the ③RAO 30° and ④LAO 45° demonstrating final positioning of the atrial lead in the Bachmann bundle region. EGM, electrogram; EGM A, atrial lead electrogram; EGM RV, right ventricular lead electrogram; RAO, right anterior oblique; LAO, left anterior oblique.

## Data Availability

The data underlying this article are available within the article.

## Notes

**Consent:** Written informed consent was obtained from the patient for publication of this case report and any accompanying images in accordance with COPE guidelines.
